# Genetic Determinants for Body Iron Store and Type 2 Diabetes Risk in US Men and Women

**DOI:** 10.1371/journal.pone.0040919

**Published:** 2012-07-16

**Authors:** Meian He, Tsegaselassie Workalemahu, JoAnn E. Manson, Frank B. Hu, Lu Qi

**Affiliations:** 1 Department of Nutrition, Harvard School of Public Health, Boston, Massachusetts, United States of America; 2 Institute of Occupational Medicine, Tongji Medical College, Huazhong University of Science and Technology, Wuhan, Hubei, China; 3 Channing Laboratory, Department of Medicine, Brigham and Women’s Hospital and Harvard Medical School, Boston, Massachusetts, United States of America; 4 Department of Epidemiology, Harvard School of Public Health, Boston, Massachusetts, United States of America; 5 Division of Preventive Medicine, Department of Medicine, Brigham and Women’s Hospital and Harvard Medical School, Boston, Massachusetts, United States of America; German Diabetes Center, Leibniz Center for Diabetes Research at Heinrich Heine University Duesseldorf, Germany

## Abstract

**Background:**

High body iron store has been associated with an increased risk of type 2 diabetes (T2D); it remains unknown whether the genetic variants related to body iron status affect T2D risk. We aimed at comprehensively investigating the associations between the genetic variants related to body iron status and the T2D risk.

**Methodology/Principal Findings:**

Six common SNPs related to body iron status from recent genome-wide association (GWA) studies were determined in the Nurses’ Health Study (NHS; 1,467 diabetic cases and 1,754 controls) and the Health Professionals Follow-up Study (HPFS; 1,124, diabetic cases and 1,298 controls). Plasma levels of ferritin, soluble transferrin receptor (sTfR), and transferrin were measured in NHS. Significant associations were observed for loci in *TPMRSS6* with sTfR (*P = *3.47×10^−6^), *TF* with transferrin (*P* = 0.0002 to 1.72×10^−10^); and *HFE* with ferritin (*P = *0.017 to 1.6×10^−8^), sTfR (*P = *0.007 to 7.9×10^−6^), and transferrin (*P = *0.006 to 0.0007). The six SNPs together explained 5.7%, 2.7%, and 13.3% of the variation in plasma levels of ferritin, sTfR, and transferrin. After adjustment for the conventional risk factors, the T allele of SNP rs855791 in the *TPMRSS6* gene was significantly associated with a 19% decreased risk of T2D (OR = 0.81; 95% CI = 0.66–0.98; *P = *0.03) in men. Multiple tests attenuated this significant association to null. No associations were observed in women. SNPs at *HFE* and *TF* were not associated with diabetes risk in either sex. Dietary iron intake did not modify the associations of the newly identified loci with diabetes risk.

**Conclusions/Significance:**

The newly identified iron-related SNP rs855791 in *TPMRSS6* was nominally associated with a decreased risk of T2D in men but not in women. The apparent differences by gender warrant further study.

## Introduction

Iron is a redox-active transitional metal and a strong pro-oxidant that can catalyze the formation of free radicals and subsequent the production of reactive oxygen species (ROS), which have been implicated in the etiology of type 2 diabetes (T2D) [1], [2]. Prospective epidemiology studies have shown consistent associations between body iron store, assessed by serum ferritin or transferrin receptors to ferritin ratio, and an increased risk of T2D [3]–[7].

Sequence variations in *HFE* gene have been associated with excess body iron store [8], [9]. In several recent genome-wide association (GWA) studies, variants in transferrin (*TF*) and *TMPRSS6* genes were also found to be related to body iron status [9], [10]. Given the observed relation between body iron store and diabetes risk, one may hypothesize that the body iron store-associated genetic variants may predispose to an increased risk of T2D. Recently one study investigated the associations between the gene polymorphisms in transferrin receptor gene and T2D [11], however, no studies have comprehensively examined the associations between the newly identified variants for body iron status and T2D.

Therefore, in the present study we aimed to examine the associations of the reported genetic variants for body iron store (ferritin, soluble transferrin receptor [sTfR], and transferrin) with T2D risk in two nested case-control studies. In our previous study, we found dietary heme iron intake modified the genetic effects of *HFE* variants on T2D risk [12]. In this study, we expanded to assess the interactions between dietary heme iron intake and other iron-associated SNPs in relation to T2D risk.

## Results

### Baseline Characteristics of Diabetic Cases and Controls

**Table 1** presents the demographic and characteristics of participants at baseline. T2D cases had a significantly higher BMI and higher prevalence of family history of diabetes than controls. Also, T2D cases engaged in less physical activity and were more likely to smoke than controls in both men and women. Among women, T2D cases were more likely to be postmenopausal. T2D cases in men and women both consumed more heme iron than controls. In NHS, the levels of ferritin, sTfR and transferrin were higher in cases than those in controls (*P*<0.05). There were no significant differences in use of iron supplement between cases and controls in both NHS and HPFS (*P = *0.13 and 0.31 respectively).

**Table 1:** Risk factor characteristics of T2D cases and controls at baseline in NHS and HPFS*.

Variables	NHS			HPFS		
	T2D (n = 1467)	Controls (n = 1754)	*P* Value†	T2D (n = 1124)	Controls (n = 1298)	*P* Value†
Age, y	43.5(6.7)	43.1 (6.8)	0.07	55 (8.6)	55 (8.4)	0.65
BMI, kg/m^2^	27.4 (5.0)	23.5 (3.9)	<0.0001	27.8 (4.0)	25.0 (2.7)	<0.0001
Family history of diabetes, %	49.5	22.1	<0.0001	33.4	13.6	<0.0001
Current smoking, %	29.4	20.9	<0.0001	12.1	7.6	<0.0001
Alcohol intake, g/d	4.4 (9.1)	6.6 (10.0)	<0.0001	11.2 (16.2)	12.1 (15.9)	0.19
Physical activity ‡	3.7 (2.8)	4.1 (2.9)	0.0005	14.6 (19.0)	21.1 (25.2)	<0.0001
Current PMH users, %	29.2	28.9	0.90	–	–	–
Postmenopausal, %	35.7	30.6	0.002	–	–	–
P:S ratio	0.34 (0.12)	0.35 (0.14)	0.26	0.55 (0.19)	0.58 (0.21)	0.004
*Trans*-fat intake, g/d	4.1 (1.3)	4.0 (1.3)	0.11	2.9 (1.0)	2.8 (1.1)	0.15
Cereal fiber intake, g/d	2.5 (1.5)	2.6 (1.6)	0.05	5.6 (3.3)	6.3 (4.8)	0.0001
Total energy intake, kcal/d	1611 (520)	1572 (488)	0.04	2100 (652)	2083 (619)	0.49
Total iron intake (mg/d)	17.5 (10.9)	17.8 (11.1)	0.51	20.0 (10.3)	20.7 (11.2)	0.13
Heme iron intake (mg/d)	1.12 (0.35)	1.04 (0.35)	<0.0001	1.29 (0.42)	1.15 (0.42)	<0.0001
Use of iron supplement, %	3.71	4.87	0.13	2.12	2.80	0.31
Ferritin (ng/ml)	105.2(101.2)	72.0 (72.1)	<0.0001	–	–	–
sTfR (mg/L)	3.38 (1.08)	3.19 (1.07)	0.006	–	–	–
Transferrin (mg/dl)	275.8 (45.6)	264.7(45.4)	0.01	–	–	–

* Values are means (SD) unless otherwise indicated. BMI  =  body mass index; PMH  =  post-menopausal hormone.

† Test of differences between cases and controls: χ2 for categorical and T-tests for continuous variables.

‡ Metabolic equivalent task hours/wk for men in HPFS and hours/wk for women in NHS.

### Associations between SNPs in *TPMRSS6*, *HFE*, and *TF* Genes and Body Iron Status

SNP rs855791 in *TPMRSS6* gene was significantly associated with high sTfR levels and sTfR : ferritin ratio (*P = *3.47×10^−6^ and 0.01) and marginally associated with low ferritin levels (*P = *0.068), but was not associated with transferrin levels (*P = *0.13). Both rs1799945 and rs1800562 in *HFE* gene were associated with ferritin (*P = *0.017 and 1.6×10^−8^), sTfR (*P = *7.9×10^−6^ and 0.007), and transferrin (*P = *0.006 and 0.0007) levels. SNPs of rs3811647 and rs1799852 in *TF* gene were associated with transferrin levels (*P = *1.72×10^−10^ and 0.0002.); However, SNP rs2280673 in *TF* gene was not associated with transferrin levels but was significantly associated with low sTfR: ferritin ratio (*P = *0.01) (**Table 2**). The six SNPs together explained 5.7%, 2.7%, 13.3%, and 3.9% of variations of plasma levels of ferritin, sTfR, and transferrin, and sTfR : ferritin ratio, respectively.

**Table 2:** Associations between body iron status and the SNPs in *TPMRSS6*, *HFE*, and *TF* genes in NHS.

SNPs*	Gene	Ferritin (ng/ml)			sTfR (mg/L)			sTfR: ferritin ratio			Transferrin (mg/dl)		
		β	se	*P*	β	se	*P*	β	se	*P*	β	se	*P*
rs855791 T/C	*TPMRSS6*	−6.41	3.21	0.068	0.17	0.04	3.47E-6	16.5	7.0	0.01	4.33	2.85	0.13
rs1799945 C/G	*HFE*	−8.82	4.27	0.017	0.20	0.05	7.9E-6	24	9.5	0.04	11.43	3.87	0.006
rs1800562 A/G	*HFE*	43.4	6.06	1.6E-8	−0.13	0.07	0.007	−22.3	13.4	0.08	−16.38	4.46	0.0007
rs3811647 A/G	*TF*	4.41	3.33	0.42	−0.03	0.03	0.65	−5.7	7.4	0.58	17.64	2.89	1.72E-10
rs1799852 T/C	*TF*	−5.59	5.12	0.18	0.04	0.06	0.72	17.1	11.2	0.12	−13.17	4.50	0.0002
rs2280673 A/C	*TF*	2.31	3.54	0.27	−0.06	0.04	0.24	−19.6	7.8	0.01	2.32	3.16	0.51

* the first allele of each SNP is effect allele.

### Body Iron Status-associated Genetic Variants and T2D Risk

**Table 3** presents the genotype frequency of the six SNPs in cases and controls respectively and the associations between the variants related to body iron status and T2D risk. The minor allele frequency of the six SNPs in the present study was similar as those reported (http://www.ncbi.nlm.nih.gov/snp). T allele of SNP rs855791 in *TPMRSS6* gene was nominally and significantly associated with a decreased risk of T2D in HPFS (OR = 0.81; 95% CI = 0.66–0.98; *P = *0.03) after adjustment for covariates; however, the SNP was not associated with T2D in the NHS. Because the body iron stores especially ferritin levels can be influenced by menopause status [13], we analyzed the associations of SNP rs855791 with T2D stratified by menopause status in NHS. We found that carriers of T allele did not significantly associate with risk of T2D in pre-menopause women (OR = 1.18; 95% CI = 0.93–1.51; *P = *0.17) and in post-menopause women (OR = 0.92; 95% CI = 0.65–1.30; *P = *0.63); and the interaction with menopause status was not significant (*P = *0.24). No significant associations were found for other five SNPs with T2D risk. After multiple tests, the association between the SNP rs855791 with T2D risk was not significant. Exclusion of iron supplement users did not materially change the associations between these SNPs and the risk T2D in both NHS and HPFS.

**Table 3:** Associations of SNPs in *TPMRSS6*, *HFE*, and *TF* genes with the risk T2D in NHS and HPFS.

SNPs	Genotype	NHS						HPFS					
		T2D n (%)	Controls n (%)	Crude OR (95% CI)	*P*	Adjusted OR* (95% CI)	*P*	T2D n (%)	Controls n (%)	Crude OR (95% CI)	*P*	Adjusted OR* (95% CI)	*P*
rs855791	CC	447(30)	551(31)	1.0	–	1.0	–	362(32)	369(28)	1.0	–	1.0	–
	CT	737(50)	872(50)	1.04 (0.89–1.22)	0.61	1.08 (0.88–1.32)	0.49	555(49)	670(52)	0.84 (0.70–1.01)	0.07	0.82 (0.67–1.01)	0.06
	TT	282(20)	329(19)	1.06 (0.86–1.29)	0.59	1.15 (0.88−1.50)	0.31	207(18)	259(20)	0.82 (0.65–1.03)	0.09	0.77 (0.59–1.00)	0.05
	CT+TT	1019(70)	1201(69)	1.05 (0.9–1.22)	0.56	1.10 (0.90–1.33)	0.36	762(67)	929(72)	0.84 (0.70–0.99)	0.04	0.81 (0.66–0.98)	0.03
	Alleles												
	C	1631(56)	1974(56)	1.0	–	–	–	1279(57)	1408(54)	1.0	–	–	–
	T	1301(44)	1530(44)	1.03 (0.93–1.14)	0.57	–	–	969(43)	1188(46)	0.90 (0.80–1.01)	0.06	–	–
rs1799945	CC	1024(70)	1223(70)	1.0	–	1.0	–	806(73)	926(71)	1.0	–	1.0	–
	CG	401(27)	478(27)	1.00 (0.86–1.17)	0.98	0.99 (0.80–1.21)	0.89	291(25)	334(26)	1.00 (0.83–1.20)	0.99	0.98 (0.79–1.21)	0.84
	GG	42(3.0)	53(3.0)	0.95 (0.63–1.43)	0.79	1.03 (0.60–1.80)	0.91	26(2.0)	38(3.0)	0.79 (0.47–1.31)	0.35	0.63 (0.34–1.16)	0.14
	CG+GG	443(30)	531(30)	1.00 (0.86–1.16)	0.96	0.99 (0.81–1.21)	0.92	317(27)	372(29)	0.98 (0.82–1.17)	0.81	0.94 (0.77–1.16)	0.57
	Alleles												
	C	2449(83)	2924(83)	1.0	–	–	–	1903(85)	2196(84)	1.0	–	–	–
	G	485(17)	584(17)	0.99 (0.87–1.13)	0.90	–	–	343(15)	410(16)	0.97 (0.83–1.13)	0.66	–	–
rs1800562	GG	1294(88)	1538(88)	1.0	–	1.0	–	995(89)	1176(91)	1.0	–	1.0	–
	GA	167(11)	203(11)	0.98 (0.79–1.22)	0.84	0.99 (0.75–1.31)	0.94	121(11)	115(9.0)	1.24 (0.95–1.63)	0.11	1.34 (0.99–1.81)	0.06
	AA	6(0.5)	13(0.7)	0.55 (0.21–1.45)	0.23	0.63 (0.20–2.05)	0.45	7(0.6)	7(0.5)	1.18 (0.41–3.38)	0.76	1.13 (0.35–3.67)	0.84
	GA+AA	173(11.5)	216(11.7)	0.95 (0.77–1.18)	0.65	0.97 (0.74–1.27)	0.82	128(11.6)	122(9.5)	1.24 (0.95–1.61)	0.11	1.33 (0.99–1.78)	0.06
	Alleles												
	G	2755(94)	3279(93)	1.00	–	–	–	2111(94)	2467(95)	1.0	–	–	–
	A	179(6.0)	229(7.0)	0.93 (0.76–1.14)	0.48	–	–	135(6.0)	129(5.0)	1.22 (0.95–1.57)	0.11	–	–
rs3811647	GG	648(44)	787(45)	1.0	–	1.0	–	492(44)	586(45)	1.0	–	1.0	–
	GA	643(44)	768(44)	1.02 (0.88–1.18)	0.82	0.99 (0.82–1.20)	0.92	486(43)	575(44)	1.01 (0.85–1.19)	0.94	1.01 (0.83–1.23)	0.90
	AA	176(12)	199(11)	1.07 (0.86–1.35)	0.54	1.02 (0.76–1.38)	0.89	146(13)	136(10)	1.28 (0.98–1.66)	0.07	1.30 (0.96–1.74)	0.09
	Alleles												
	G	1939(66)	2342 (67)	1.00	–	–	–	1470(65)	1747(67)	1.0	–	–	–
	A	995(34)	1116(33)	1.08 (0.97–1.20)	0.17	–	–	778(35)	847(33)	1.09 (0.97–1.23)	0.15	–	–
rs1799852	CC	1189(81)	1429(81)	1.0	–	1.0	–	901(80)	1043(80)	1.0	–	1.0	–
	CT	260(18)	310(18)	1.01 (0.84–1.21)	0.93	1.02 (0.80–1.30)	0.86	213(19)	240(19)	1.03 (0.84–1.26)	0.80	1.09 (0.86–1.37)	0.49
	TT	18(1.0)	15(0.8)	1.44 (0.72–2.87)	0.30	1.31 (0.55–3.08)	0.54	10(0.8)	14(1.0)	0.83 (0.37–1.87)	0.65	1.11 (0.45–2.71)	0.82
	CT+TT	278(19)	325(19)	1.03 (0.86–1.23)	0.76	1.04 (0.82–1.31)	0.76	223(20)	254(20)	1.02 (0.83–1.24)	0.87	1.09 (0.86–1.37)	0.47
	Alleles												
	C	2638(90)	3168(90)	1.0	–	–	–	2015(90)	2326(90)	1.0	–	–	–
	T	296(10)	340(10)	1.05 (0.89–1.23)	0.60	–	–	233(10)	268(10)	1.00 (0.83–1.21)	0.97	–	–
rs2280673	CC	579(39)	743(43)	1.0	–	1.0	–	452(40)	532(41)	1.0	–	1.0	–
	CA	696(47)	797(45)	1.12 (0.97–1.30)	0.13	1.15 (0.94–1.40)	0.17	515(46)	604(47)	1.00 (0.85–1.19)	0.97	0.92 (0.76–1.12)	0.40
	AA	192(13)	214(12)	1.15 (0.92–1.44)	0.22	1.25 (0.93–1.68)	0.14	157(14)	161(12)	1.15 (0.89–1.48)	0.28	1.08 (0.80–1.44)	0.63
	Alleles												
	C	1854(63)	2283(65)	1.0	–	–	–	1419(63)	1668(64)	1.0	–	–	–
	A	1080(37)	1225(35)	1.09 (0.98–1.20)	0.12	–	–	829(37)	926(36)	1.05 (0.94–1.18)	0.40	–	–

* Adjusted for age, BMI (<23.0, 23.0–24.9, 25.0–29.9, 30.0–34.9, or ≥35.0 kg/m^2^ ), family history of diabetes (yes, no), smoking (never, past, current), alcohol (nondrinker or drinker [0.1–4.9, 5.0–9.9, 10.0–14.9 or ≥15.0 g/day]), menopausal status [pre- or post-menopausal (never, past, or current hormone use); women only], quintiles of physical activity (metabolic equivalent task hours/wk for men in HPFS, hours/wk for women in NHS), and quintiles of energy adjusted P:S ratio, *trans*-fat and cereal fiber intakes.

**Figure 1:**
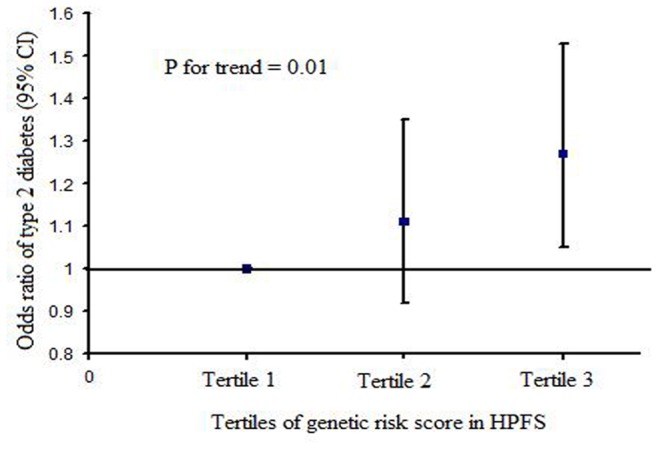
Associations between tertiles of genetic risk score and type 2 diabetes risk in HPFS.

We further examined the combined effects of the iron-related SNPs on T2D risk. The results indicated that the GRS was associated with higher risk of T2D in men. Subjects in the highest tertile of the GRS had a 27% increased risk of T2D (95% CI: 1.05–1.53; *P = *0.01), compared with those in the lowest tertile (**Figure 1**). The OR (95% CI) for T2D associated with each point scored, corresponding to one risk allele, was 1.06 (1.01–1.12; *P = *0.03). Adjusted for lifestyle risk factors except BMI did not alter the results. Further adjustment for BMI attenuated the associations to marginal significance. The GRS was not associated with T2D risk in women (data not shown).

### Interactions with Heme Iron Intakes in Relation to T2D Risk

In our previous analyses, we found genetic variants in *HFE* gene interacted with heme iron intakes in relation to T2D risk [12]. In the present study we examined the interactions of heme iron intake with SNPs in other two loci *TPMRSS6* and *TF*, but did not find significant interactions in either men or women (data not shown).

## Discussion

In this study we confirmed that the recently identified SNPs in *TPMRSS6* and *TF* genes were associated with plasma biomarkers for body iron store, sTfR and transferrin [9], [10]. We found SNP rs855791 in *TMPRSS6* gene was associated with lower risk of T2D in men but not in women. Multiple tests attenuated this significant association to null. No significant interactions between the newly identified SNPs and heme iron intake were observed in relation to T2D risk.

The iron-T2D association has been extensively investigated. It was found that 53–80% of patients with haemochromatosis, an autosomal-recessive disorder with iron overload, developed diabetes [14]. Evidences from the epidemiological studies in the general population also support the relation between excess body iron store and an increased risk of T2D [3]–[7]. Genetic variants in *HFE* genes, C282Y (rs1800562) and H63D (rs1799945), have been related to high body iron store [8], [9]. However, in our previous meta-analysis, we found that these variants were not associated with T2D risk [12].

A recent GWA study found a nonsynonymous variant rs855791 in *TMPRSS6* gene was associated with several indicators of body iron status including serum iron, transferrin saturation, and ferritin [10]. TMPRSS6, a transmembrane serine protease, plays a key role in iron homeostasis by inhibiting the production of hepatic hepcidin [15], and then influences the intestinal iron absorption and the release of iron from cellular stores [16]. We demonstrated the T allele of SNP rs855791 was associated with lower ferrtin and higher sTfR levels and sTfR : ferritin ratio and this allele was related to a decreased risk of T2D only in men. However, after multiple tests, this association was not significant. This might be due to the small sample size and the weak effects of the SNP on T2D risk. Menstruation in women causes iron loss, which might explain the difference in the genetic associations with T2D between men and women. In fact, in our present study the post-menopause women had higher levels of plasma ferritin (111.0±99.8 vs 79.1±82.9 ng/ml; *P*<0.001) than pre-menopause women. Among post-menopause women the T allele was not significantly associated with risk of T2D; however, the effect direction of this allele was in line with the observations in men.

The significant associations between *TF* variants and plasma transferrin levels were consistent with the results in a GWA study [9]. Transferrin has been associated with insulin resistance, through increasing the rate of adipocyte lipolysis and circulating free fatty acid [17]. However, we did not find associations between the *TF* SNPs and T2D risk.

The null-associations between body iron-associated genetic variants and T2D can be explained in several ways. First, the six SNPs explained only a very small proportion of the variance in body iron store. Therefore the weak association between the genetic markers and T2D would be expected. Second, although both the NHS and HPFS dataset had more than 80% power to detect SNPs with odds ratio >1.4, given an α of 0.05 and allele frequency of 0.10, our study might be underpowered for detecting weaker associations. The NHS dataset only has 60% power to detect genetic odds ratio of 1.2 for SNPs with MAFs equal to 0.10, at the 5% significance level; for the HPFS dataset the power is 47%. Finally, inflammation might influence the findings in the present study. Ferritin is believed to be influenced by inflammation. sTfR might also be regulated by inflammatory cytokines [18] and insulin [19]. Although some studies found that the iron-diabetes associations were independent of inflammation, it is still remained to investigate in future studies. Especially, there is evidence indicating that subunits of ferritin may be a better biomarker than ferritin for assessing body iron status and that the ratio of H:L-ferritin subunit levels can help to distinguish the inflammation-induced or excessive iron-induced elevation of ferritin [20].

Our data did support an additive effect of the variants at the three loci *HFE*, *TMPRSS6,* and *TF* on T2D risk. The GRS combining these genetic variants was associated with an increased risk of T2D in men. Adjustment for the potential risk factors especially BMI attenuated the associations of the iron-related GRS and T2D risk, indicating that BMI might partially explain these associations. However, the underlying mechanism remained to be elucidated in further studies. We did not find the associations of GRS and T2D risk in women. It might be due to the gender difference as discussed above.

In our earlier analyses, we reported that the genetic variants in *HFE* gene interacted with heme iron intake in relation to T2D [12]. However, we did not find significant interactions between the newly identified SNPs of *TMPRSS6* and *TF* genes and heme iron on diabetes risk. These observations suggest that the effects of the SNPs in *TMPRSS6* and *TF* genes on T2D risk are not modified by dietary iron intakes. The failure in identifying gene-diet interactions may also due to the low power of the study.

Our study samples are highly homogeneous by only including Caucasians with European ancestry. Our GWA analyses indicate that the associations in these samples are not affected by population stratification [21]. It remains to be determined whether our findings are applicable to other populations. Another issue is that some of our control subjects may have undiagnosed diabetes, which might bias our results toward the null.

However, because the participants in our study are health professionals and our previous study also showed that the undiagnosed diabetes prevalence was low (approximately 2%) [22], it might not appreciably affect our results.

In summary, we demonstrated associations between newly identified genetic markers in *TPMRSS6* and *TF* genes and body iron stores, and found that SNP rs855791 in *TPMRSS6* was nominally associated with a decreased risk of T2D in men. Future studies are needed to investigate the association of these iron related variants and T2D risk in larger studies and in different populations, as well as to elucidate potential gender difference in the genetic effects.

## Methods

### Study Population

The NHS was established with the recruitment of 121,700 female registered nurses (aged 30–55 years) and residing in 11 large U.S. states completed a mailed questionnaire on their medical history and lifestyle characteristics [23]. Beginning in 1980, dietary information has been updated every 2 to 4 years using validated semi-quantitative food frequency questionnaires (FFQs). Every two years, follow-up questionnaires have been sent to update information on potential risk factors and lifestyle information and to identify newly diagnosed cases of T2D and other diseases [24]. The HPFS began in 1986 when 51 529 male U.S. health professionals (aged 40–75 years) answered a detailed questionnaire that included a comprehensive diet survey, and items on lifestyle practice and medical history [25]. The cohort is followed through biennial mailed questionnaire. Dietary information is updated every four years [26]. Blood was collected from a total of 32 826 NHS members between 1989 and 1990 and from 18 159 HPFS members between 1993 and 1999.

Subjects for the present study were selected from those who provided blood samples using a nested case-control study design [27], [28]. Demographics and health status of participants who provided blood samples were generally similar to those who did not. T2D cases were identified by self-report methods that were confirmed with a validated supplementary questionnaire [29], [30]. For cases before 1998 cycle, we used the National Diabetes Data Group criteria [31] to define diabetes. The validity of this method has been established [32]. We used the 1997 ADA diagnostic criteria for diabetes diagnoses from 1998 onwards [33]. Among the diabetes cases, a total of 98% of self-reported diabetes cases were confirmed by medical records review in both NHS and HPFS cohorts [29]. Controls were free of diabetes at the time the case diagnosis and remained unaffected through follow- up till 2006. All participants provided written informed consent, and the study was approved by the Human Research Committee at the Brigham and Women’s Hospital, Boston.

### Assessment of Covariates

Information about medical history, anthropometrical data, lifestyle factors, and family history of diabetes in first-degree relatives was derived from the baseline questionnaires [25], [34]. BMI was calculated as weight in kilograms divided by the square of height in meters (kg/m^2^). For men, physical activity was expressed as metabolic equivalent task (MET) hours of moderate to vigorous exercise per week and was calculated by using the reported time spent on various activities, weighting each activity by its intensity level. For women, physical activity was expressed as hours per week because MET was not measured at baseline in the NHS. Self-administered questionnaires about body weight and physical activity have been validated as described previously [35], [36]. Energy-adjusted intakes of heme iron (mg/d) were calculated based on FFQs. We used the average intakes of baseline dietary heme iron from the 1980 and 1984 questionnaires in NHS and 1986 and 1990 questionnaires in HPFS for gene-dietary heme iron interaction analysis. The correlation coefficient for energy-adjusted total dietary iron intake from the FFQ and two 1-week diet records spaced 6 months apart was 0.60 after adjustment for within-person variability in daily intake [24]. In addition, heme iron consumption based on the FFQ has been shown to be related to plasma ferritin levels in NHS [37].

### DNA Extraction, Single Nucleotide Polymorphisms (SNPs) Selection and Genotyping Methods

In the NHS nested case-control study, 67.8% blood samples were obtained after 8-hour overnight fasting; in HPFS, it is 59.0%. DNA was extracted from the buffy coat fraction of centrifuged blood using a QIAmp blood kit (Qiagen, Chatsworth, CA). A genome-wide scan was conducted using the Affymetrix Genome-Wide Human 6.0 array and the Birdseed calling algorithm [21]. The quality control of genotyping and related analysis methods for our GWA study were described previously [21]. In this study, six SNPs related to iron stores, including H63D (rs1799945) and C282Y (s1800562) in *HFE* gene, rs855791 in *TPMRSS6* gene [10], and rs3811647, rs1799852, and rs2280673 in *TF* gene [9] were extracted from the genome-wide scans. The six SNPs did not significantly deviate from Hardy-Weinberg equilibrium (HWE) in controls in either NHS or HPFS after adjustment for multiple testing (*P*>0.008: α = 0.05/6 SNPs).

### Determinant of Plasma Levels of Biochemical Markers in NHS

The iron status biomarkers were measured in NHS. Blood samples in NHS were collected in1989 and 1990 [38]. Concentrations of ferritin and transferrin receptors were measured by a particle-enhanced immunoturbidimetric assay using the Hitachi 911 analyzer (Roche Diagnostics, Indianapolis, IN) [12]. Transferrin was measured by an immunoturbidimetric assay using the Hitachi 917 analyzer and Roche Diagnostics reagents (Indianapolis, IN). To minimize bias and interassay variation, study samples were selected from randomly ordered case-control pairs in measurement. The coefficients of variation for ferritin, transferrin receptors, and transferrin were 3.75%, 8.4%, and 6.0% respectively.

### Statistical Analyses

Deviations from HWE were assessed by chi-square tests. We used logistic regression to estimate ORs for T2D risk, adjusting for age (in years), BMI (<23.0, 23.0–24.9, 25.0–29.9, 30.0–34.9, or ≥35.0 kg/m^2^ ), family history of diabetes (yes, no), smoking (never, past, current), alcohol intake (nondrinker or drinker [0.1–4.9, 5.0–9.9, 10.0–14.9, or ≥15.0 g/day]), menopausal status [pre- or post-menopausal (never, past, or current hormone use); women only], quintiles of physical activity, and quintiles of energy adjusted P:S ratio, *trans*-fat and cereal fiber intakes. Power calculations were performed using Quanto 1.2.3 (http://hydra.usc.edu/gxe).

A genetic risk score (GRS) was calculated by summing up the number of alleles of the six variants associated with higher levels of markers of body iron. Interactions between SNPs and the dietary heme iron intake were assessed by entering the cross-product of the two variables into the model. To normalize the distributions, plasma ferritin, sTfR, and transferrin were inverse normal transformed. We used R^2^, which was the difference of model sum of squares between models with and without the SNPs of interest divided by the corrected total sum of squares of the full model, to calculate the percentage of the variance of the biomarkers that were explained by the SNPs. Because multiple variants were chosen to be investigated in the present study, we used the Bonferroni’s correction to do the multiple comparisons [39]. The SAS statistical package was used for the analyses (SAS, version 9.0 for UNIX). All *P* values are two sided.
